# Fusion oncogenes in rhabdomyosarcoma: model systems, mechanisms of tumorigenesis, and therapeutic implications

**DOI:** 10.3389/fonc.2025.1570070

**Published:** 2025-06-17

**Authors:** Chinmay S. Sankhe, Lisa Hall, Genevieve C. Kendall

**Affiliations:** ^1^ Center for Childhood Cancer Research, The Abigail Wexner Research Institute, Nationwide Children’s Hospital, Columbus, OH, United States; ^2^ Division of Hematology and Oncology, Nationwide Children’s Hospital, Columbus, OH, United States; ^3^ Department of Pediatrics, The Ohio State University College of Medicine, Columbus, OH, United States

**Keywords:** pediatric sarcomas, PAX3/7::FOXO1, VGLL2::NCOA2, skeletal muscle, oncogenic drivers, animal models

## Abstract

Rhabdomyosarcoma (RMS) contributes to 3% of all childhood cancers with roughly 400-500 cases diagnosed each year in the United States. The World Health Organization classifies rhabdomyosarcoma into four histological subtypes which include alveolar, embryonal, spindle-cell and pleomorphic. The primary genetic drivers in a subset of alveolar and spindle-cell histological subtypes are gene fusions. This review explores the fusion oncogenes identified in RMS such as *PAX-*and *NCOA2-*based fusions, along with discussing studies defining fusion oncogene biology and tumorigenic mechanisms. Focus areas include data around transformation events and progression along with dysregulated biological processes. Furthermore, we summarize model systems, ranging from cell to animal models, that have been implemented to study fusion oncogenes identified in RMS. With the constant identification of novel fusion oncogenes, this review also emphasizes the need for genetically characterizing RMS tumors and rapidly developing new model systems. These models are critical to study fusion oncogene activity and to delineate key regulatory players and potential therapeutic targets that suppress tumorigenesis. The identification of RMS fusion oncogenes and integration with animal and cell culture models will help identify conserved molecular targets, optimize therapeutic approaches, and ultimately improve clinical outcomes for children with RMS.

## 1 Introduction

Rhabdomyosarcoma (RMS) is a rare soft-tissue sarcoma that affects predominantly children but can also present in adulthood [reviewed in (1)]. RMS is associated with significant diagnostic and therapeutic challenges due to the diverse genetic drivers and varying disease aggressiveness. Understanding the biological mechanisms driving RMS subtypes will help identify therapeutic opportunities with the goal of improving clinical outcomes. The most aggressive forms of RMS are genetically driven by fusion oncogenes that engage different transcriptional signatures, which ultimately converges on tumors with molecular features of arrested skeletal muscle differentiation. Traditionally, fusion-positive RMS has focused on *PAX-*based fusion oncogenes of *PAX3::FOXO1* and *PAX7::FOXO1.* However, new RMS fusion oncogenes are being rapidly identified from clinical sequencing efforts with little to no knowledge of their tumorigenic mechanisms. Here, fusion-positive refers to the PAX3/7::FOXO1 fusions and fusion driven refers to the collection of fusions found in RMS. In this review, we will discuss RMS fusion oncogenes and summarize studies of disease-driving mechanisms and functional targets. First, for context, we briefly describe clinical presentations of RMS. Next, we detail RMS fusion oncogenes and segregate the discussion based on if the fusion partners are *PAX3/7* or *NCOA2*. We summarize key studies describing fusion oncogene function and how it contributes to RMS initiation and progression. Finally, we focus on approaches to functionally validate and model fusion oncogenes by describing experimental strategies and available genetic animal tumor models. Such integrated bench to bedside approaches are critically needed for fusion driven pediatric sarcoma patients. The goal of this review is to discuss progress and identify remaining challenges in our understanding of fusion oncogene-driven RMS, providing a basis for future studies to support identifying novel therapeutic targets.

### 1.1 Clinical presentation

RMS accounts for 50% of all pediatric soft-tissue sarcoma cases and the tumors express proteins such as myogenin (MyoG), desmin and MyoD that are indicative of immature skeletal muscle [reviewed in (1–3)]. RMS presents in soft tissues in body regions such as the head, neck, chest, bladder, prostate, arms, legs, and trunk, with specific subtypes having more common presentation sites. RMS typically metastasizes to the lung, bone marrow and lymph nodes (4). Roughly 400-500 new cases of RMS are diagnosed in the United States annually. Of these cases, 59% occur in children (<19 years) and 41% occur in adults (>19 years) (1, 2, 5). In children, more than 50% of cases are seen in the first decade of life (6). A subset of these patients will have a germline cancer predisposition syndrome such as Li-Fraumeni or DICER1 (7, 8). Five-year survival rates vary based on risk stratification, a combination of site, nodal involvement, metastases, age, surgical resection, and FOXO1 fusion presence (9). These risk stratification factors for RMS patients, including the presence of the PAX3/7::FOXO1 fusion, were also the basis of a clinical trial developed by the European pediatric soft tissue sarcoma study group (10). These rates decrease from 70-90% in low-risk pediatric groups to 20-30% in high risk. Adults have poorer outcomes compared to the pediatric groups, largely based on the presence of metastases at diagnosis. Adult 5 year overall survival is 20% (5, 11). Overall survival also varies with histological subtype (embryonal, alveolar, spindle-cell, and pleomorphic) (12). Current treatments for RMS include chemotherapy, radiation therapy, surgery, or multimodal therapy; the specific treatment plans are often guided by risk stratification and recently have included molecular diagnostics once available [reviewed in (1)]. There are no therapies that directly target the primary oncogenic drivers of the disease.

**Figure 1:**
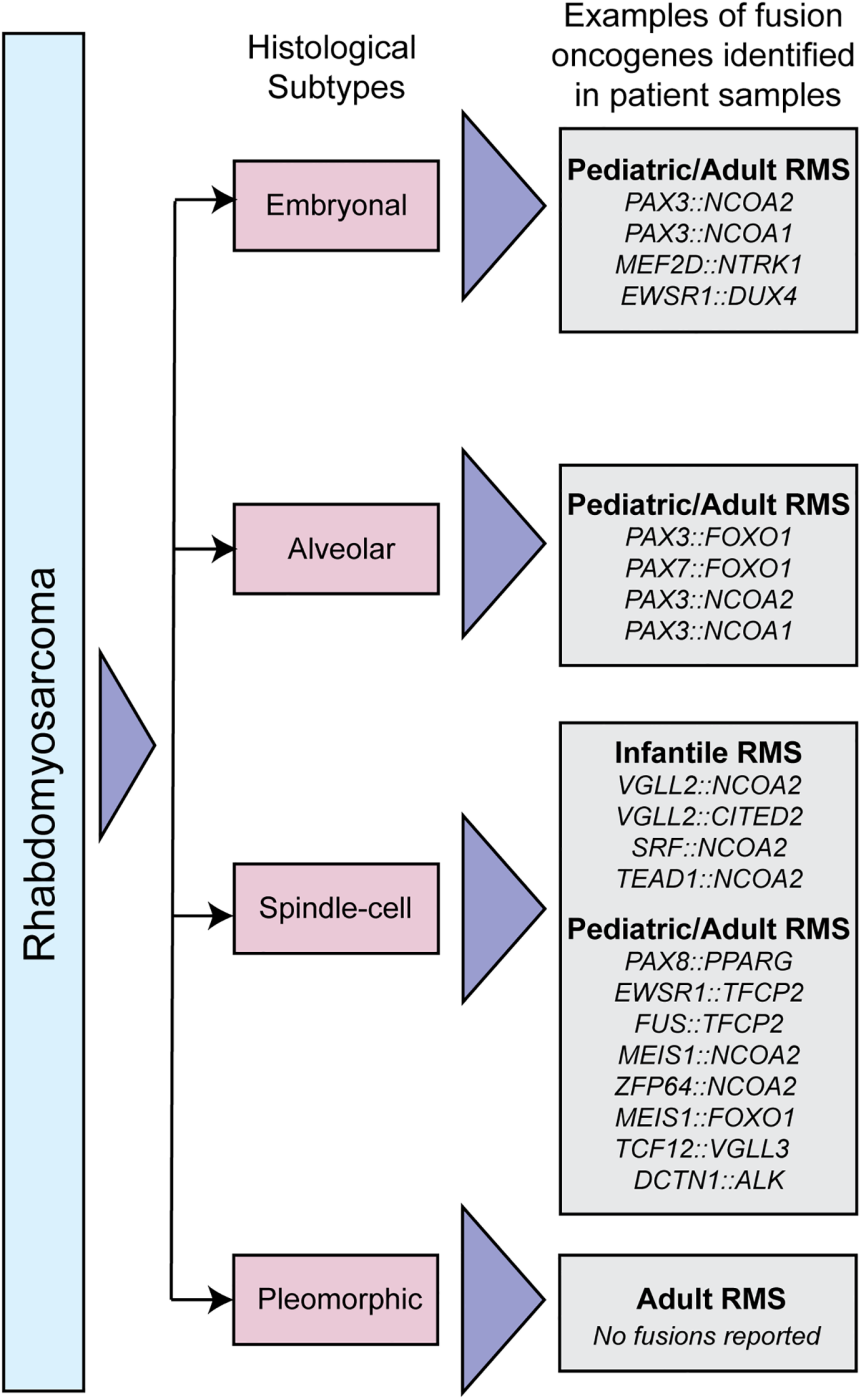
Classification of RMS subtypes. Schematic depicting different histological RMS subtypes, and examples of fusion oncogenes in each subtype that have been identified in patient samples.

RMS has four histological subtypes, some of which are driven by gene fusions. Embryonal RMS is the most common subtype contributing to 60% of all RMS cases and also has the most favorable prognosis [reviewed in (13)]. Most embryonal RMS cases occur in children under 10 years of age and originate in the head and neck, prostate, urinary bladder, and abdomen; this includes tissues of the ear, tongue, and nasopharynx [reviewed in (2, 14)]. Embryonal RMS is not genetically fusion driven, but rare fusions have been described, including PAX3::NCOA1/2. Alveolar RMS is a more aggressive subtype of RMS that affects primarily teens and young adults; it contributes to roughly 20% of all RMS cases. This subtype is associated with unfavorable prognosis and commonly originates in regions such as limbs, trunks, head, and neck [reviewed in (2, 15)]. The most common genetic drivers are PAX3/7::FOXO1. Spindle-cell or sclerosing RMS is a rare RMS subtype that accounts for 10% of all RMS cases and has a broader presentation spectrum. It affects both children (and infants) and adults, with a more favorable prognosis in children. This subtype typically presents in the head, neck, and brain region (16, 17). In infants, spindle cell RMS is typically driven by NCOA2 fusions. Pediatric and adult cases of spindle cell RMS are also driven by a collection of fusion oncogenes, a subset which is detailed in **Figure 1**. Pleomorphic RMS is an aggressive RMS subtype that often metastasizes to distant tissues. It is associated with 10% of all RMS cases and occurs in adults with an age group spanning from the early twenties to 80. This subtype is associated with unfavorable prognosis and is found in the chest, abdomen, shoulder, and lower extremities (18–21), and is not known to be driven by gene fusions. Understanding the genetics of the disease, especially the presence or absence of gene fusions, guides clinical treatment plans (among other factors) and has prognostic importance. Here, we will focus on the fusion-positive RMS and fusion driven spindle cell/sclerosing RMS.

## 2 PAX-based fusion oncogenes

Approximately 80% of alveolar RMS are genetically driven by a paired box (*PAX*) gene-associated chromosomal translocations (22–24). In 60% of alveolar RMS cases, the *PAX3* DNA-binding domain on chromosome 2 is fused to the forkhead box O1 (*FOXO1*) transactivation domain on chromosome 13 (referred to as PAX3::FOXO1). In 20% of alveolar RMS cases, the *PAX7* gene on chromosome 1 is fused to the *FOXO1* gene on chromosome 13 (referred to as PAX7::FOXO1) (2, 24–26). In 20% of alveolar RMS cases, there is no fusion oncogene that has been detected to date. This subset is indistinguishable from embryonal RMS with no fusion oncogenes in terms of gene expression signatures and clinical outcomes (27).

Normally, PAX3/PAX7 and FOXO1 both act as transcription factors with critical roles in neural crest specification and differentiation, skeletal muscle development, and in cell proliferation processes. PAX3 directly regulates the transcriptional activity of *MYF5, MYOD1*, and *FGFR4*, which play a role in skeletal muscle development. It also regulates *MITF, TYRP1, RET, TBX2, NGN2, HES1, and NRCAM*, which contribute to neural development. Additionally, PAX3 plays a role in chromatin structure regulation (28). PAX7 is a myogenic transcription factor important for renewal and maintenance of satellite cells seen in postnatal skeletal muscles. Pax7 deficient mice were significantly smaller than wildtype and had reduced muscle mass indicating that knockout of Pax7 inhibited satellite cell-mediated growth of skeletal muscle (29, 30). Importantly, PAX3 and PAX7 are in-part functionally redundant but not completely. In a study investigating the functional roles of Pax3 and Pax7 in limb muscle development, the *Pax3* gene was replaced by *Pax7* in mice. Pax7 was able to compensate for the loss of Pax3 for neural tube closure, neural crest cell development and migration; however, these mice had a loss of forelimb muscle development (31). FOXO1 is a member of the forkhead box protein O family of transcription factors and is expressed in most muscle types. It regulates muscle regulatory roles of growth, glucose metabolism, and differentiation (32). Subcellular localization of FOXO1 is crucial for regulating myogenic differentiation; nuclear export of FOXO1 is especially important for early skeletal muscle differentiation (33). One hypothesis is that the fusion oncogenes retain the activity of their normal fusion partners.

**Figure 2:**
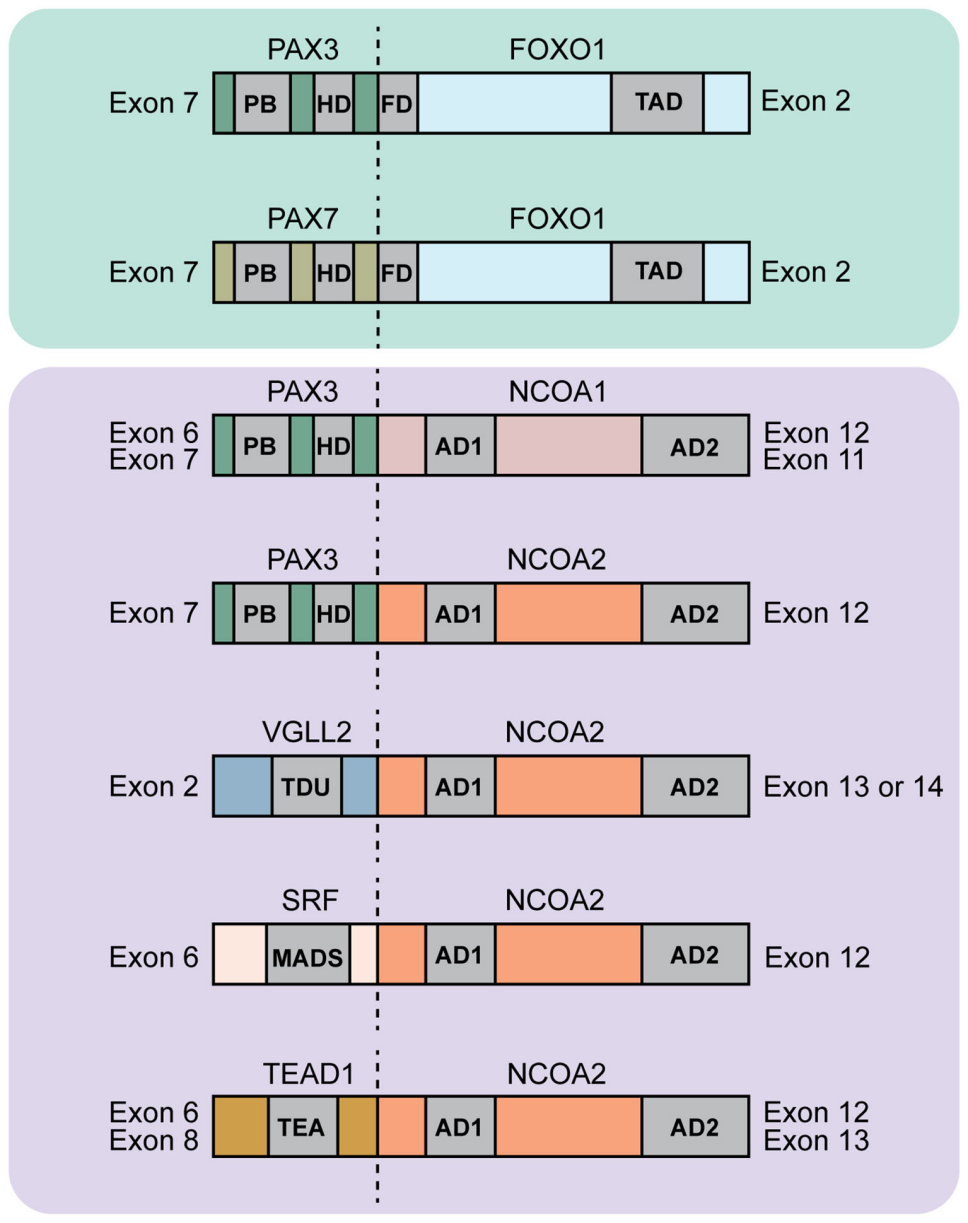
Diagram of protein domains found in rhabdomyosarcoma fusions. Shown are a subset of FOXO1 and NCOA2 fusions. The dotted lines indicate the fusion junction. For the PAX*-*based fusion oncogenes the abbreviations indicate the following: Paired box gene 3/7 (PAX3/7), forkhead box O1 (FOXO1), PB: paired box domain, HD: homeodomain, FD: forkhead domain, TAD: Transactivation domain. PAX3/PAX7::FOXO1 fuses exon 7 of PAX3/7 to exons 2-3 of FOXO1 [ref (34, 35)]. For the NCOA-based fusion oncogenes the abbreviations indicate the following: Paired box gene 3 (PAX3), nuclear receptor coactivator 1/2 (NCOA1/2), AD1, AD2: activating domains 1 and 2 of NCOA genes. PAX3::NCOA1 fuses either exons 1-6 (type 1) or exons 1-7 (type 2) of PAX3 to exons 12-20 (type 1) or exons 11-20 (type 2) respectively of NCOA1. Type 1 has 894 amino acids with 319 amino acids of PAX3 and 575 amino acids of NCOA1. Type 2 has 1026 amino acids with 391 amino acids of PAX3 and 635 amino acids of NCOA1. PAX3::NCOA2 fuses exons 1-7 of PAX3 to exons 12-23 of NCOA2. This fusion protein is 1057 amino acid long with 391 amino acids of PAX3 and 666 amino acids of NCOA2 [ref (36)]. For the VGLL2::NCOA2 fusion oncogene the abbreviations indicate the following: Vestigial-like family member 2 (VGLL2), TDU: Tondu domain. VGLL2::NCOA2 fuses exons 1-2 of VGLL2 to exon 13 or 14-23 of NCOA2 [ref (37, 38)]. For the SRF::NCOA2 fusion oncogene, abbreviations indicate the following: Serum response factor (SRF), MADS: MCM1, AGAMOUS, DEFICIENS, and SRF domain. SRF::NCOA2 fuses exon 6 of SRF to exon 12 of NCOA2 [ref (39)]. For the TEAD1::NCOA2 fusion oncogene, abbreviations indicate the following: TEA domain transcription factor 1 (TEAD1) also called transcriptional enhancer factor (TEF1). TEAD1::NCOA2 fuses either exon 8 (type 1) or exon 6 (type 2) of TEAD1 to exon 13 (type 1) or exon 12 (type 2) respectively of NCOA2 [ref (39, 40)].

The PAX3/7::FOXO1 fusions juxtapose the PAX3 or PAX7 5’ DNA binding paired box and paired-type homeodomains to the FOXO1 3’ transactivation domain (**Figure 2**). PAX3::FOXO1 and PAX7::FOXO1 fusions likely function differently. This is supported from both clinical and experimental data. Clinically, PAX3::FOXO1 RMS patients have reduced overall survival compared to PAX7::FOXO1 patients (22, 24, 41–43). Further, genetic animal model studies show PAX3 and PAX7 are not functionally redundant or fully compensatory (31), and a direct comparison of PAX3::FOXO1 and PAX7::FOXO1 activity highlights key differences, suggesting shared and divergent functions (44). In patient tumors, PAX7::FOXO1 often has copy number amplification which is not true for PAX3::FOXO1 (45). One interesting note is that molecularly, PAX3::FOXO1 patient tumors have enhanced staining for proliferation marker MIB1, along with higher apoptosis, as compared to PAX7::FOXO1 tumors (46). In the subsequent sections, we summarize the studies examining the neomorphic functions of PAX3::FOXO1, which is more prevalent, aggressive, and more studied than PAX7::FOXO1.

### 2.1 PAX3::FOXO1 activity in cellular contexts

PAX3::FOXO1 has been studied in diverse contexts with functions ranging from transcriptional to epigenetic. In NIH3T3 cells, transient transfection of *PAX3::FOXO1* and *PAX3* identified that PAX3::FOXO1 is a more potent transcriptional activator than PAX3 (47). This suggests PAX3::FOXO1 can lead to enhanced or persistent activation of normal PAX3 target genes. In human SaOS-2 and U2-OS osteosarcoma cells, ectopic PAX3 expression initiated mesenchymal-epithelial transition, yet PAX3::FOXO1 expression had an even more pronounced effect (48). This study also identified distinct PAX3::FOXO1 gene signatures compared to PAX3, suggesting that not all targets overlap with PAX3 and that PAX3::FOXO1 has neomorphic activity. A study using alveolar RMS cells (RH30 and RH4) found that PAX3::FOXO1 can inhibit the activity of the endogenous FOXO1 transcription factor, as cells transfected with siRNA inhibiting *PAX3::FOXO1* had an increase in FOXO1 protein levels. Knocking down PAX3::FOXO1 in human RMS cells resulted in decreased cell proliferation, lower motility, increased protein levels of Desmin and Myosin Heavy Chain (MHC), down-regulation of mRNA levels of *MYOD1, MYF5, MRF4*, and increased myogenin protein levels, highlighting the importance of PAX3::FOXO1 in tumor maintenance and in suppressing myogenic differentiation (49).

In fibroblast and epithelial-like cells, studies have supported varying roles of PAX3::FOXO1 in regulating proliferation and provided a platform for comparing PAX3/7::FOXO1 activity. Transduction of retroviral PAX3::FOXO1 into chicken embryo fibroblasts increased growth and colony formation in soft agar (50). In a separate study, PAX3::FOXO1 expression in mouse fibroblasts supported a transformed phenotype with increased contractility and anchorage-independent growth (51). The level of PAX3::FOXO1 protein can also induce discrete phenotypes, with low PAX3::FOXO1 expression in mouse fibroblasts resulting in transformation, while high expression of PAX3::FOXO1 supported growth inhibition (52). In human foreskin fibroblasts, PAX3::FOXO1 and PAX7::FOXO1 expression had discrete transcriptomic landscapes with higher deposition of H3K27 acetylation in the case of PAX7::FOXO1 binding compared to PAX3::FOXO1 (44). A recent publication reports a dual inducible model system to study PAX3::FOXO1 and HES3 genetic cooperation using HEK293T cells. Cells expressing only PAX3::FOXO1 formed fewer spheres compared to dual-induced cells with both PAX3::FOXO1 and HES3 expression. This supports that HES3 promotes PAX3::FOXO1 transformation *in vitro* (53).

PAX3::FOXO1’s oncogenic activity have also been investigated in mesenchymal stem cells and skeletal muscle myoblast cells, which have been proposed as a potential RMS cell(s) of origin. In mouse mesenchymal stem cells (MSCs), expression of PAX3::FOXO1 and PAX7::FOXO1 resulted in enhanced growth and alveolar RMS tumors in mouse allografts with cooperating mutations. Required cooperating mutations include activated RAS, *tp53* mutation and expression of SV40 early region (54). C2C12 mouse myoblast cells can tolerate PAX3::FOXO1 expression, and exhibit increased proliferation and inhibition of myogenic differentiation (55). In primary human myoblasts, PAX3::FOXO1 expression enhanced proliferation and drove cell growth past the senescence stage. This increase in cell proliferation was accompanied by a reduction in protein levels of tumor suppressor CDKN2A (p16^INK4A^) (56). PAX3::FOXO1 can also act as a pioneer factor as shown biochemically in RMS cell lines and genomically in a human inducible myoblast model. PAX3::FOXO1 binds to regions of closed chromatin in addition to accessible chromatin, allowing it to control distinct cell-fate decisions (57). PAX3::FOXO1 pioneering activity and functional consequences were also shown with an *in vivo* embryonic zebrafish model. These studies identified that PAX3::FOXO1 utilizes partial homeobox motif recognition to bind to closed chromatin. Whereas, binding to its composite paired-box/homeobox motif resulted in increased chromatin accessibility and activation of neural gene signatures (58).

In immortalized human myoblast cells, PAX3::FOXO1 expression promoted proliferation, inhibited myogenic differentiation, and formed xenografts in SCID mice, albeit slow growing. PAX3::FOXO1 activity was augmented by MYCN overexpression, and xenografting these cells resulted in a faster tumor growth rate than PAX3::FOXO1 alone (59). In an inducible *PAX3::FOXO1* human myoblast system, the fusion-oncogene cooperated with MYCN to inhibit differentiation and promote proliferation during transformation; however, in this context, PAX3::FOXO1 is not required for tumor recurrence (60). In a follow-up study, PAX3::FOXO1 genetically cooperates with transcriptional target, FGF8 (with constitutive MYCN expression), to enhance cell proliferation and tumor growth (61).

Taken together, these studies suggest that PAX3::FOXO1 has diverse regulatory functions depending on the cell type, thereby highlighting the need to understand the potential RMS cell(s) of origin and how PAX3::FOXO1 might function in those contexts.

### 2.2 PAX3::FOXO1 targets

PAX3::FOXO1 binds and activates target genes that make up its core regulatory circuitry (62). Furthermore, studies support that PAX3::FOXO1 utilizes super enhancers such as FOXO1 cis-regulatory domains in association with other transcription factors of MYCN, MYOD and myogenin to inhibit skeletal muscle differentiation (63, 64). This results in locking RMS cells in an undifferentiated myogenic-like stage that is highly proliferative. PAX3::FOXO1-target genes likely have diverse pro-tumorigenic functions such as stimulating proliferation and invasion, inhibiting differentiation, and promoting cancer cell survival by repressing tumor suppressors. Understanding PAX3::FOXO1 targets and their functional requirements for the disease could represent potential therapeutic opportunities. **Table 1** outlines key genes that are upregulated by ectopic PAX3::FOXO1 expression.

**Table 1:** Key differentially regulated genes following ectopic *PAX3::FOXO1* expression in cells.

PAX3::FOXO1 induction	Cell type	Key regulated genes	Method used for identifying gene targets	Reference
Retroviral transduction	NIH3T3 cells	*MYOD, MYOG, SIX1, SLUG*, growth factor *IGF2*	cDNA microarray	Khan, J. et al., 1999 (65)
Stable transfection	ERMS cell line RD	*ITM2A, BVES, TGFA, FLT1*	PCR-based cyclic amplification and selection of targets	Barber, T.D. et al., 2002 (66)
Retroviral transduction	ERMS cell line RD	*GREM1, DAPK1, MYOD1*	Quantitative RT-PCR	Eun Hyun, A.H.N, 2013 (67)
Stable transfection	293T cells	*TFAP2B*	Comparative expression profiling	Ebauer, M. et al., 2007 (68)
Adenoviral transduction	Human osteosarcoma cell lines	*CNR1, EPHA2, and EPHA4*	Microarray analysis	Begum, S. et al., 2005 (48)

Different cellular contexts modify PAX3::FOXO1 activity. For example, in NIH3T3 cells, transduction of *PAX3::FOXO1* upregulated gene expression of *MYOD, MYOG, SIX1, SLUG*, and growth factor *IGF2* (65). Given the toxicity of the fusion, another strategy is introducing PAX3::FOXO1 into an embryonal or fusion-negative RMS cell line. This approach identified activation of genes such as *ITM2A, BVES, TGFA, and FLT1*, shared gene targets of the *PAX3* gene (66). Viral transduction of *PAX3::FOXO1* into embryonal RMS cells identified direct targets including tumor suppressor gremlin 1 (*GREM1*), death-associated protein kinase-1 (*DAPK1*), and *MYOD1* (67). A parallel study using inducible PAX3::FOXO1 expressed in human fusion-negative embryonal cell line RD confirmed this finding along with identifying target genes involved in apoptosis, development, and signal transduction (69). Comparative expression profiling was utilized to determine PAX3::FOXO1 specific genes, and *TFAP2B* was identified as the direct target regulating PAX3::FOXO1’s anti-apoptotic roles (68). Examining the global gene signatures of alveolar and embryonal RMS resulted in the identification of genes that are uniquely overexpressed in alveolar RMS: cannabinoid receptor 1 (*CNR1*), *PIPOX*, *DCX, ABAT, JAKMIP2, NRCAM*, *DKFZp762M127*, and *FOXF1* (70). Using chromatin immunoprecipitation (ChIP), this study found that PAX3::FOXO1 directly binds to the promoter of *CNR1, EPHA2*, and *EPHA4*, thereby, regulating their expression (48). Another study implemented ChIP sequencing on RMS PAX3::FOXO1-positive cell lines and fusion-negative cells transfected with *PAX3::FOXO1* and found that the binding sites of PAX3::FOXO1 are majorly located distal to the transcription start sites and highly correlated with genes upregulated in PAX3::FOXO1-positive cells. This study also identified genes *FGFR4* and *IGF1R* to be direct targets of PAX3::FOXO1 (71). In addition, ChIP sequencing on RH4 RMS cells revealed PAX3::FOXO1 genome-wide binding patterns were different than core regulatory transcription factors of MYCN, MYOG, MYOD1, and that PAX3::FOXO1 binding sites were adjacent to repressive histone marks H3K9me3 and H3K27me3 (57). These data suggest that not all the PAX3::FOXO1 ChIP binding sites are conserved across cell lines, indicating different RMS tumors might have unique biology.

PAX3::FOXO1 plays a dual role of regulating induction and inhibition of myogenesis. NIH3T3 cells transduced with PAX3*::*FOXO1 had elevated MyoD and myogenin protein expression; however, the cells did not differentiate or form myotubes, suggesting that PAX3::FOXO1 inhibited complete myogenic differentiation. This inhibition of myogenic differentiation was mediated by fibroblast growth factor receptors (FGFRs) (72). In another study examining PAX3/7::FOXO1’s role in myogenesis, expression of PAX3/7::FOXO1 inhibited terminal differentiation and suppressed the *MyoD*-target gene, *MyoG*. However, PAX3/7::FOXO1 did not affect *MyoD* transcriptional activity or binding at the *MyoG* promoter. Instead, PAX3/7::FOXO1 decreased histone H4 acetylation and reduced RNA polymerase II binding at the *MyoG* promoter, indirectly impacting MyoD’s normal function (73). In a study investigating genetic cooperation of PAX3::FOXO1 and the cannabinoid receptor 1 (CNR1) in a panel of RMS cell lines, *CNR1* mRNA expression levels were only upregulated in alveolar RMS cells. CNR1 did not contribute to the PAX3::FOXO1 cell proliferation and differentiation, but it was essential for regulating PAX3::FOXO1*-*induced invasion and metastasis, suggesting that CNR1 could be considered a therapeutic opportunity (74).

PAX3::FOXO1 also has epigenetic targets that support its oncogenic activity. For example, jumonji and AT-rich interaction domain-containing 2 (*JARID2)* gene levels are increased in PAX3::FOXO1 fusion-positive RMS patient tumors, with JARID2 expression being dependent on PAX3::FOXO1. *JARID2* knockdown decreased cell proliferation, increased cell elongation, and activated protein expression of myogenin and myosin light chain, both differentiation markers. Mechanistically, *JARID2* bound the promoter region of myogenin and myosin light chain, and together with Polycomb Repressive Complex 2 (PRC2) complex, increased H3K27Me3 deposition leading to their repression and maintenance of an undifferentiated cell state (75). KDM4B, a histone lysine demethylase, also plays a role in regulating PAX3::FOXO1 activity in cell culture models. Induction of PAX3::FOXO1 in two systems (an immortalized human myoblast and human fusion-negative embryonal RD cells) led to elevated levels of KDM4B, elongated morphology, and transformation. Depletion of KDM4B in RH30 and Rh41 resulted in reduced cell growth (76), decreased colony formation, and delayed tumor formation in xenografts (77). PAX3::FOXO1 and KDM4B form a complex and regulate the expression of MYOD1 (77). In a recently published study, another histone lysine demethylase, KDM3B, was shown to be crucial for the tumorigenic activity of PAX3::FOXO1. KDM3B suppression inhibits PAX3::FOXO1 activity, with the readouts being cell growth *in vitro* and *in vivo* (78).

These data suggest that PAX3::FOXO1 acts as a transcriptional activator regulating genes involved in inhibiting terminal skeletal muscle differentiation, promoting proliferation and invasion, and driving RMS tumorigenesis. PAX3::FOXO1 targets such as IGF1R, CNR1, JARID2, KDM4B and KDM3B could be further explored as therapeutic approaches for PAX3::FOXO1*-*driven RMS.

## 3 NCOA2-based fusion oncogenes

### 3.1 PAX3::NCOA1/NCOA2 fusion oncogene structure

Nuclear receptor coactivator 2 (NCOA2) is a nuclear hormone receptor and a member of the p160 steroid nuclear receptor coactivator family (39, 79). The nuclear hormone receptor plays essential roles in various cellular processes such as cell growth, differentiation, inflammatory and metabolic pathways (80, 81). NCOA2 consists of basic helix-loop-helix and Per-Arnt-Sim domain (bHLH/PAS) receptor nuclear translocator domain along with transcriptional activation domains (AD) CID/AD1 and AD2 (82). NCOA2 and its close family member, NCOA1, are common fusion partners in cancer encompassing soft tissue sarcoma, prostate and breast cancer and acute myeloid leukemia as examples (83–88). PAX3::NCOA1 and PAX3::NCOA2 fusions are found in rhabdomyosarcoma. The resulting fusion protein consisted of the paired-box and DNA binding domain of PAX3 along with the CID/AD1 domain, the Q-rich region, and the AD2 domain of NCOA1 or NCOA2 (**Figure 2**). Normally, the NCOA2 CID/AD1 domains bind CBP/p300, whereas the AD2 domain binds with CARM1/PRTM1, among other interactions (82, 89, 90). Reverse transcriptase polymerase chain reaction (RT-PCR) analysis of patient tumor samples revealed two different *PAX3::NCOA1* translocations with fusion of the *PAX3* exon 7 to *NCOA1* exon 11 and fusion of the *PAX3* exon 6 to *NCOA1* exon 12 (36). The *PAX3* exon 6 fusion to *NCOA1* exon 12 was also identified by an earlier study by Wachtel M. et al. (91). In the case of *PAX3::NCOA2*, the fusion incorporates the *PAX3* exons 1-7 and *NCOA2* exons 12-23, both of which contain the protein domains previously highlighted.

Functional studies of these PAX3::NCOA1/2 fusion oncogenes have been performed in fibroblast and myoblast cell culture systems. Transfection of NIH3T3 mouse fibroblast cells with the fusion oncogenes resulted in colony formation, whereas deletion of CID/AD1 and the AD2 domain of the NCOA proteins resulted in a significant decrease in colony formation capacity (36). Another study identified PAX3::NCOA2 in a patient tumor diagnosed with embryonal RMS. Transfection of *PAX3::NCOA2* in C2C12 cells resulted in increased proliferation and higher motility as compared to the control-transfected cells; however, the increase in proliferation and motility was still less compared to cells transfected with *PAX3::FOXO1*. *PAX3::NCOA2-*transfected C2C12 cells also had reduced capacity for myogenic differentiation in culture. This study also showed that PAX3::NCOA2 expressing C2C12 cells were able to form allograft tumors in nude mice at a slower rate than PAX3::FOXO1 allograft tumors (92). These studies indicate that fusion oncogenes with one shared fusion partner can both be transforming with different capacity; suggesting, the 3’ fusion partner supports unique tumorigenic mechanisms.

### 3.2 Infantile/pediatric rhabdomyosarcoma NCOA2 fusion oncogene structure

Characterizing pediatric and adult spindle-cell RMS specimens via next-generation RNA sequencing led to identification of two *NCOA2*-based RMS fusion oncogenes (39). The first NCOA2 fusion was with the serum response factor (*SRF*) gene with exon 6 of *SRF* fused to the exon 12 of *NCOA2* (**Figure 2**). SRF is a transcription factor that is highly expressed in skeletal muscle and regulates genes involved in muscle development and differentiation (93). The second NCOA2 fusion was with the TEA domain transcription factor 1 (TEAD1) with exon 8 of *TEAD1* fused to the exon 13 of *NCOA2* (**Figure 2**). An Archer Anchored Multiplex PCR analysis on a 16-month-old spindle-cell RMS patient identified exon 6 of *TEAD1* fused with exon 12 of *NCOA2* (40). TEAD1 is expressed in skeletal muscle and regulates genes involved in metabolism and developmental processes (94). In a follow-up study characterizing spindle-cell infantile/pediatric RMS tumor specimens, additional fusions were identified including VGLL2::NCOA2, VGLL2::CITED2, TEAD1::NCOA2, and SRF::NCOA2. Further analysis revealed that intron 3 of *VGLL2* fused with exon 2 of *CITED2* in four infantile/pediatric spindle-cell RMS cases, while exon 2 of *VGLL2* fused with exon 14 of *NCOA2* in two RMS cases (37, 95) (**Figure 2**). Normally, vestigial-like family member-2 (*VGLL2*) promotes skeletal muscle differentiation via translocating to the nucleus and interacting with the TEAD1 transcription factor (96). VGLL2 is also co-expressed with myogenin in differentiating muscle cells (97). Thus, the VGLL2::NCOA2 fusion might disrupt and co-opt the muscle differentiation cascade for tumorigenesis.

NCOA2 is a common fusion partner, especially in sarcoma. **Table 2** summarizes a subset of the different NCOA2-based fusions that have been identified to date. Some fusions, like MEIS1::NCOA2, were identified in patients with spindle-cell RMS, intraosseous spindle-cell RMS, and spindle-cell sarcoma of the kidney in adults, highlighting that the same fusion can have different presentations (98–100). This is likely a collaborative process between fusion acquisition and cell of origin.

**Table 2:** NCOA2-based fusion oncogenes identified in different cancer subtypes.

Sr. No	Cancer subtype	Fusion oncogene	Reference
1.	Infantile/Pediatric Spindle-cell RMS	*VGLL2::NCOA2* *TEAD1::NCOA2* *SRF::NCOA2*	Alaggio, R. et al., 2016, Mosquera, J.M. et al., 2013 (37, 39)
2.	Spindle-cell RMS	*MEIS1::NCOA2* *ZFP64::NCOA2*	Smith, B.F. et al., 2023, Montoya-Cerrillo, D.M. et al., 2020, Argani, P. et al., 2018, Dehner, C.A. et al., 2023 (98–101)
3.	Biphenotypic sinonasal sarcoma, Alveolar/Embryonal RMS	*PAX3::NCOA2* *PAX3::NCOA1*	Sumegi, J. et al., 2010, Le Loarer, F. et al., 2019 (36, 102)
4.	Acute leukemia with coexpression of T-lymphoid and myeloid	*NCOA2::ETV6*	Strehl, S. et al., 2008 (103)
5.	Acute myeloid leukemia	*NCOA2::MOZ*	Troke, P.J. et al., 2006, Deguchi, et al., 2003, Yin, H. et al., 2007 (85–87)
6.	Soft tissue angiofibroma	*NCOA2::AHRR*	Jin, Y. et al., 2012 (104)
7.	Mesenchymal chondrosarcoma	*HEY1::NCOA2*	Wang, L. et al., 2012 (88)
8.	Fibroblastic spindle-cell mesenchymal neoplasms	*CTCF::NCOA2 CRTC1::NCOA2 CTCF::NCOA3*	Bakhswin, A. et al., 2024 (105)
9.	Uterine sarcoma	*GREB1::NCOA2*	Brunetti, M. et al., 2018 (106)
10.	Colorectal cancer	*LACTB2::NCOA2*	Yu, J. et al., 2016 (107)

## 4 Modeling fusion oncogene driven rhabdomyosarcoma

To understand fusion oncogene activity and guide targeted treatment, it is essential to functionally validate their activity using model systems including cell and animal models. Clinical sequencing efforts are rapidly identifying new fusion oncogenes in RMS with limited models to mechanistically study them. We have summarized some examples of the different fusion oncogenes found in RMS and some sarcoma subtypes in **Table 3**, many of which have no cell or animal models of the disease. This section will discuss the different types of model systems (cell and animal based) that are used for functional genomics of RMS-specific fusion oncogenes. Every model has its benefits and challenges, and the choice of model depends on the research question being asked. Additional factors to consider when deciding on a model include generation time, efficiency, relevance to human disease, cost, experience, and infrastructure and ease of experimental setup.

**Table 3:** Examples of fusion oncogenes in RMS and other sarcoma subtypes.

Sr. No	Cancer subtype	Fusion oncogene	Reference
1.	Alveolar RMS	*PAX3::FOXO1*	Barr, F.G. et al., 1993, Galili, N. et al., 1993, Shapiro, D.N. et al., 1993 (26, 108, 109)
2.	Alveolar RMS	*PAX7::FOXO1*	Davis, R.J. et al., 1994 (110)
3.	Alveolar RMS	*PAX3::AFX(FOXO4)*	Barr, F.G. et al., 2002 (111)
4.	Alveolar RMS, Biphenotypic sinonasal sarcoma	*PAX3::MAML3*	Dermawan, J.K. et al., 2024, Wang, X. et al., 2014 (112, 113)
5.	Biphenotypic sinonasal sarcoma, Alveolar/Embryonal RMS	*PAX3::NCOA2* *PAX3::NCOA1*	Sumegi, J. et al., 2010, Le Loarer, F. et al., 2019 (36, 102)
6.	Embryonal RMS without tp53 mutations	*MEF2D::NTRK1*	Li, N.M. et al., 2023 (114)
7.		*EWSR1::DUX4*	Sirvent, N. et al., 2009 (115)
8.	Congenital fibrosarcoma	*ETV6::NTRK3*	Bourgeois et al., 2000 (116)
9.	Small round cell sarcomas	*EWSR1::PATZ1* *FUS::NFATC2* *EWSR1::TFCP2* *FUS::TFCP2 CIC::DUX4/FOXO4/NUTM1 BCOR::CCNB3/MAML3*	Watson, S. et al., 2018 Kawamura-Saito, M. et al., 2006, Szuhai, K. et al., 2009, Pierron, G. et al., 2012 (95, 117–119)
10.	Infantile/Pediatric Spindle-cell RMS	*VGLL2::NCOA2* *SRF::NCOA2* *TEAD1::NCOA2* *VGLL2::CITED2*	Alaggio, R. et al., 2016, Mosquera, J.M. et al., 2013 (37, 39)
11.	Spindle-cell RMS	*PAX8::PPARG*	Rakheja, D. et al., 2022 (120)
12.	Spindle-cell RMS in bone and soft tissue	*EWSR1::TFCP2 MEIS1::NCOA2* *ZFP64::NCOA2 MEIS1::FOXO1* *TCF12::VGLL3* *DCTN1::ALK* *FUS::TFCP2*	Dehner, C.A. et al., 2023 (101)

### 4.1 Cell culture and PDX models

#### 4.1.1 PAX3::FOXO1 cell models

There are a variety of cell culture strategies and patient-derived cell lines used to study fusion oncogene-driven RMS [reviewed in (121)]. Patient-derived CW9019 harbors the PAX7::FOXO1 translocation (122) while cell lines such as KFR, RH10, RH30, and TC212 harbor the PAX3::FOXO1 fusion (121). Another RMS cell line, RUCH-2, was derived from a primary botryoid RMS patient with positive expression for protein markers of PAX3, MYF3 and MYF5, but lost expression of MYF3 and MYF5 after 3.5 months in culture. However, the cells became metastatic with increased invasiveness and had the ability to form faster tumors in nude mice. Thus, this cell line facilitated comparison between the primary and metastatic phase in RMS (123).

To complement patient cell lines, studies have also induced the expression of PAX3::FOXO1 in human immortalized myoblast cells to understand transformation and changes in proliferative state. Human skeletal muscle myoblast cells were used that had been immortalized with overexpression of hTERT and absence of Ink4a/ARF. In these myoblasts, PAX3::FOXO1 expression in combination with MYCN generated RMS-like tumors in SCID/*beige* mice, suggesting the role of these components in driving myoblast cells to a tumorigenic state (124). In several other studies, dual expression of PAX3::FOXO1 and MYCN in immortalized human myoblast cells has been shown to develop rapid tumors in SCID mice by maintaining the cells in a proliferative state and preventing cellular differentiation compared to PAX3::FOXO1 expression alone (59–61).

RMS 3D cell culture models have also been explored to better recapitulate an *in vivo* environment (125–127). In a RH28 and RH30 3D rhabdosphere model of PAX3::FOXO1-driven RMS, non-adhered spheres had increased expression of stemness markers such as SOX2, NANOG, and OCT4. The spheres could also generate tumors at a faster rate than adherent monolayer RH30 cells when injected into immunodeficient mice (125). RH30 cells have also been cultured on a collagen sponge inside a bioreactor system with perfusion flow leading to a 3D organotypic cell culture RMS model. The cells under perfusion flow exhibited higher proliferation with elevated levels of matrix metalloprotease 2 and invasive RMS protein marker of LAMA1/2 as compared to the static cultured cells (125, 127). These systems could represent cost-efficient strategies to complement *in vivo* animal modeling.

#### 4.1.2 PAX3/7::FOXO1 single cell RNA sequencing in cell and PDX models

In the past few years, there have been multiple single cell RNA sequencing transcriptomic analyses that have aided in identifying muscle-specific lineages and cell subpopulations represented in RMS. A single-cell atlas of patient tumor samples, patient-derived cell lines and xenograft models revealed that RMS tumors consist of proliferative, apoptotic and differentiating cell subpopulations along with quiescent progenitor cells. This study also found that PAX3::FOXO1 and PAX7::FOXO1 tumors have an unique neuronal cell subpopulation expressing genes such as *DCX, L1CAM, SYP* and *CHGA*. Importantly, these same neural genes were activated in response to chemotherapy suggesting they have a role in therapeutic resistance (128). Another study on RMS patient-derived xenograft cultures and alveolar RMS cell lines found three different cell states. The first subpopulation resembled early myogenic muscle stem cell-like state with high expression of cell adhesion genes. The seconds subpopulation was proliferating cells with enrichment in cell division genes, and the third subpopulation was differentiated cells with expression of myotube and terminal differentiation genes (129).

Another single cell RNA sequencing study using RMS patient-derived xenografts found similar heterogeneity in RMS tumors with a differentiated cell subpopulation expressing muscle genes of *MYLPF, ACTC1, LRNN1, TNNT3* and *TSPAN33;* progenitor cells expressing extracellular matrix genes of *MMP2*, and the majority of RMS cell states expressing *MYOD* and *DES* (130). On investigating the tumor microenvironment in RMS, this study found that the presence of differentiated macrophages in the M2 polarization state are linked with angiogenesis and suppression of inflammation. There was also a presence of dendritic and undifferentiated macrophages subpopulations in this tumor microenvironment, indicative of immune dysfunction (131).

Investigating primary embryonal and alveolar RMS patient tumor tissues using single cell RNA sequencing revealed that tumors exhibited subpopulations of paraxial mesoderm expressing *MEOX2* and *PAX3*, myoblasts expressing *MYF5* and *MSC*, and myocytes expressing *MYOG* and *MEF2C*. In addition, there were differences between alveolar RMS and embryonal RMS, where a greater percentage of tumor cells in alveolar RMS exhibited myocyte-like state expressing *MYOG*, and a lesser percentage exhibited paraxial mesoderm with *MEOX2* expression (132). Thus, RMS-based tumors are heterogenous with multiple cell subpopulations contributing to tumor-related activities that can be aligned with developmental timepoints. Further, fusion-positive RMS is less adhered to the myogenic hierarchy and tumors and PDX samples express markers of neural subpopulations.

#### 4.1.3 Rhabdomyosarcoma patient-derived xenograft and organoid models

Researchers have also developed models to better reflect the immune environment or use primary patient tumor samples. For example, a mouse xenograft system was established in an immunocompetent mouse to model immunotherapy-based approaches in RMS. This system integrated a humanized immune system into mice with a severe lack of innate immunity. To do this, human hematopoietic stem cells were transplanted into the mice followed by subcutaneously xenografting human RMS cell lines, RD or RH30. Histological analysis of the tumors revealed integration of human RMS cells with the human immune system containing few monocytes and B and T cells located in peripheral blood. This study serves as an exciting approach to evaluate immunotherapeutic strategies in RMS-based tumorigenesis (133). In another study, a pleomorphic patient-derived RMS tumor was used to develop an orthotopic xenograft model (PDOX) and subcutaneous transplant model. Tumors that formed in the PDOX model were significantly faster growing and more invasive, suggesting a malignant state, compared to the subcutaneous transplant tumors, indicative of a benign state (134).

Xenograft models have also been used to evaluate efficacy of chimeric antigen receptor (CAR)-T based approaches. For example, FGFR4 has been identified as overexpressed in RMS and this overexpression is predictive of reduced overall survival (135, 136). FGFR4 is a cell surface signaling molecule and amenable for CAR-T based targeting. As such, using *in vitro* and *in vivo* CAR-T based approaches, FGFR4 has been targeted causing specific cytotoxicity *in vitro* and decreased tumor burden *in vivo* in an intramuscular PAX3::FOXO1 xenograft model (137, 138). Further, there were few observed off-target effects such as weight loss and fur loss, and the mice tolerated the therapy. This strategy is now being explored as an antibody drug conjugate (139), and has promising translational potential for treating children with RMS.

Another approach for modeling RMS is using tumor organoid models which offers benefits of faster processing and supporting high-throughput drug screening. These organoids are formed by plating the minced patient tumors in basement membrane extract or extracellular matrix component. This study developed 19 pediatric alveolar and embryonal RMS tumor organoids that genetically and histologically resembled the original tumors (140). A limitation is being at a large medical center to obtain primary tissue for these rare diseases. Efforts have been undertaken to develop patient derived tumor organoid screening platform to test drug resistance and generate personalized therapeutic approaches in various sarcomas (141, 142). This approach can be further explored to answer biological and clinical research questions pertaining to RMS.

#### 4.1.4 Infantile/pediatric spindle cell rhabdomyosarcoma models

S-RMS1 was developed from resected fresh tumor extracted after surgery from a 4-month-old boy with infantile/pediatric spindle-cell RMS. This patient derived cell line harbored the SRF::NCOA2 fusion and expresses RMS markers of MyoD, desmin and myogenin. The cell line extensively overlapped with the tumor at diagnosis using whole genome sequencing. The study also compared this cell line with other human RMS cell lines of RH30 and RD18 and found that S-RMS1 had a lower doubling time than RH30 and RD18 along with higher mRNA transcript levels of neovascularization marker *endoglin* and *GATA-6*. Other markers such as skeletal muscle differentiation markers of *MEF2A*, *MEF2B*, *MEF2C*, and *MEF2D* in S-RMS1 were similar to the mRNA levels seen in RH30 and RD18 (143). A recent study developed a VGLL2::NCOA2 cell culture model using C2C12 mouse myoblast cells, where the cells were transfected to stably express human VGLL2::NCOA2. C2C12-VGLL2::NCOA2 expressing cells were allografted into immunodeficient mice and generated aggressive and rapid tumors compared to C2C12 pcDNA3.1 controls (38). This study found that C2C12 expressing VGLL2:NCOA2 could transform and induced a developmental gene program that highlighted potential therapeutic opportunities. This strategy was expanded upon by another group that showed that expressing the TEAD1::NCOA2 fusion in C2C12 mouse myoblast cells also generated colonies *in vitro* and tumors in a mouse allograft model. The study also demonstrated that inhibiting p300 activity via a small molecule inhibitor led to reduction in TEAD1::NCOA2 colony formation along with suppressing tumor growth in the allograft model (144).

### 4.2 Animal models

#### 4.2.1 PAX7::FOXO1 drosophila model

A drosophila model is the only animal model for PAX7::FOXO1. In this strategy, PAX7::FOXO1 is expressed in myosin heavy chain positive cells. This is lineage restricted by using a UAS/GAL4 genetic system. After crossing UAS/GAL4 flies, PAX7::FOXO1 is conditionally expressed in Drosophila syncytial myofibers. Further monitoring revealed that PAX7::FOXO1 expression resulted in the formation of nucleated cells that separated from the Drosophila syncytial myofibers and spread to non-muscular regions and the central nervous system (145). In a follow-up study utilizing the PAX7::FOXO1 Drosophila fly model as the basis for a genetic screen, the authors found that *Mef2* is a target gene of the PAX7::FOXO1 fusion and that inhibiting *Mef2* suppressed PAX7::FOXO1*-*dependent phenotypes (146).

#### 4.2.2 PAX3::FOXO1 chick neural tube model

PAX3::FOXO1 expression in neural crest cells derived from chick embryos is a strategy to understand the tumorigenic early response and transformation potential. In this system, PAX3::FOXO1 decreased expression of neurogenic markers of SOX2 and PAX6 and caused reorganization of the ventricular and mantle regions of the neural tube as compared to PAX3 overexpression and control embryos. In addition, after extracting chick neural cells from the neural tube at 48 hours post electroporation, PAX3::FOXO1 expression elevated the levels of fusion-positive RMS genes *ALK, ARHGAP25* and *FGFR4*, and transcription factors such as *EYA2, FOXF1, LMO4, MEOX1, MYOD1, PITX2, PAX2, PRDM12* and *TFAP2β*. In contrast, PAX3 overexpression did not regulate these genes. Thus, induction of PAX3::FOXO1 resulted in reprogramming chick neural cells to exhibit fusion-positive RMS-like features (147).

#### 4.2.3 Pax3::Foxo1 mouse model

The Pax3::Foxo1 mouse model was developed in 2004. This model has exons 2-3 of *Foxo1* conditionally knocked in to the endogenous *Pax3* allele after exon 7. After crossing with a Myf6-Cre driver, Pax3::Foxo1 is expressed in Myf6+ cells and the normal *Pax3* allele is disrupted. This led to the formation of 1 tumor out of 228 animals at 12 months without cooperating mutations. The addition of Ink4a/ARF (or CDKN2A) or Trp53 mutations increased Pax3::Foxo1 tumor latency and frequency (148). In a separate study credentialing the Pax3::Foxo1 mouse model, induction of Pax3::Foxo1 expression in mice along with mutations in Ink4a/ARF (or CDKN2A) or Trp53 resulted in increased tumor penetrance. This tumor model recapitulates the aggressive nature of human alveolar RMS pediatric cancer and demonstrates rapid growth, invasion, involvement of regional lymph nodes along with distant hematogenous metastasis (149). The Pax3::Foxo1 genetically engineered mouse model has been used to understand genetic cooperation in the disease including the role of Hippo signaling in regulating Pax3::Foxo1-driven RMS tumorigenesis. Hippo signaling is suppressed in alveolar RMS cell lines and tumors (150), and genetically inhibiting the Hippo/MST signaling by knocking out kinases *MST1* and *MST2 (Stk4, Stk3)*, led to more rapid *Pax3::Foxo1* tumor onset and higher penetrance. This indicates a dual regulatory mechanism where loss of MST kinases along with expression of Pax3::Foxo1 and mutational loss of *Cdkn2a* promotes more aggressive disease (151).

The Pax3::Foxo1 mouse model with inactivation of the p53 pathway has been leveraged to understand transformation capacity of a subset of myogenic and endothelial lineages. Evaluated lineages include Pax3 (MCre-Pax3), embryonic and fetal lineage (Myf6Cre), and postnatal satellite cell lineage (Pax7CreER). MCre-Pax3 and Myf6Cre resulted in RMS tumors resembling alveolar RMS histology, while Pax7CreER led to pleomorphic or spindle cell-like tumors. Notably, Myf6Cre showed the highest tumor incidence, indicating that Pax3::Foxo1 expression in specific lineages can drive more aggressive tumor behavior (152). The expression of Myf6 in this Cre driver is postulated to have both an embryonic expression with unclear timing and postnatal expression. In a recent study, expression of Pax3::Foxo1 in a Tek-Cre-driven mice endothelial lineage generated tumors similar to RMS. Pax3::Foxo1 tumors expressed desmin, MyoD1 and myogenin at the mRNA and protein level. In another lineage of *Fabp4-Cre*, induction of Pax3::Foxo1 drove high penetrance tumors that were consistent with RMS (153). These data suggest that there could be multiple cell(s) of origin outside of a myogenic lineage, and that Pax3::Foxo1 has the capacity to transdifferentiate an endothelial cell to a tumor cell expressing skeletal muscle proteins.

#### 4.2.4 PAX3::FOXO1 zebrafish model

**Figure 3:**
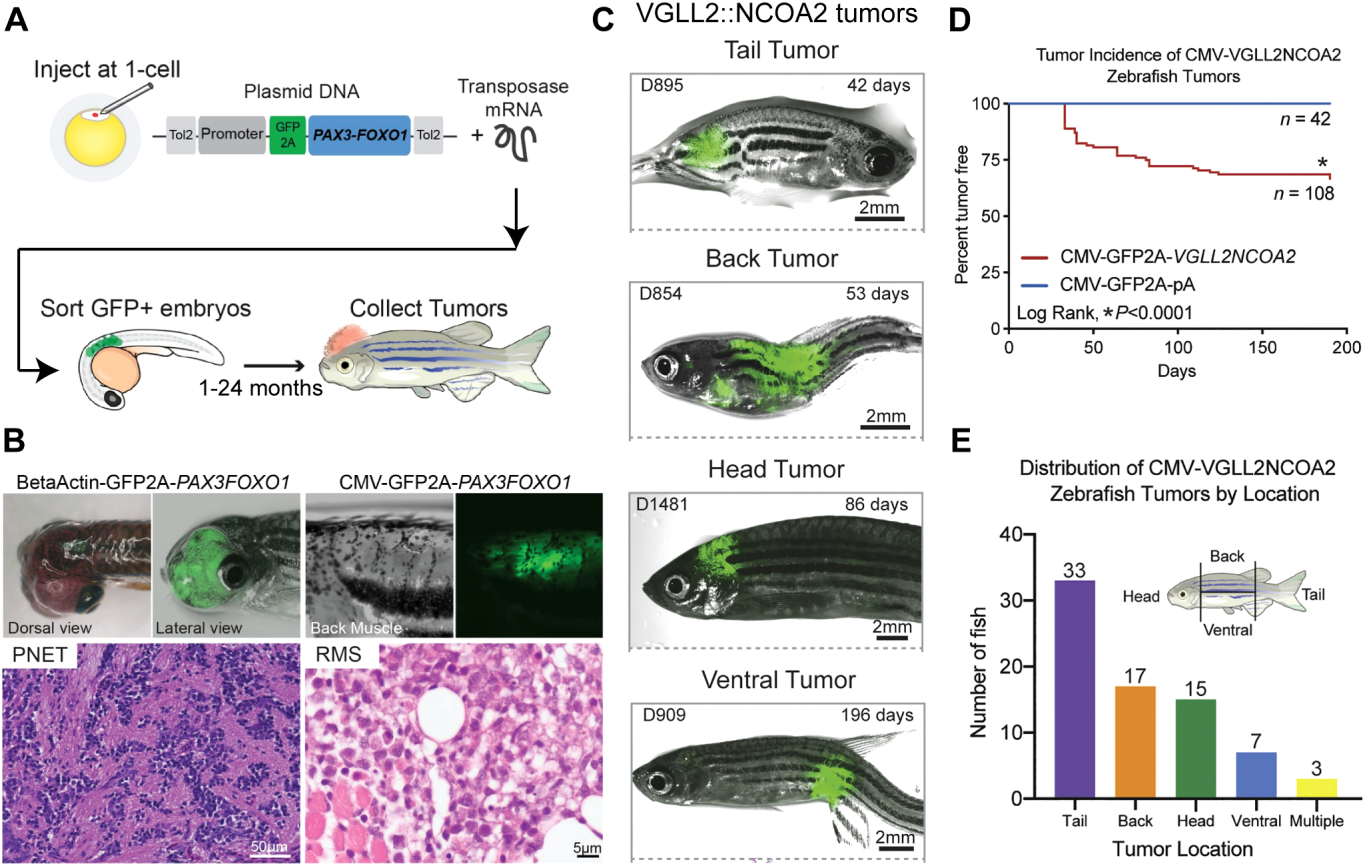
Functional genomics of rhabdomyosarcoma fusion oncogenes using zebrafish. **(A)** Schematic depicting the integration of the fusion oncogene and the viral 2A linked fluorescent protein into the Tol2 transposon backbone. Zebrafish embryos are injected at the single-cell stage and then GFP+ embryos are sorted after 24 hours and monitored for tumor formation. **(B)** The β-actin promoter driving *PAX3::FOXO1* led to formation of tumors in zebrafish consistent with primitive neuroectodermal tumors. CMV promoter driven *PAX3::FOXO1* formed RMS tumors, but required a cooperating *tp53* missense mutation. **(C)** Representative zebrafish tumors formed using the CMV promoter driven *VGLL2::NCOA2* fusion and classified by location of the tumor. **(D)** Tumor incidence curve comparing the tumor formation in VGLL2::NCOA2 injected zebrafish with the control-injected zebrafish. **(E)** Distribution of zebrafish VGLL2::NCOA2*-*injected tumors segregated as per the tumor location on the fish. Panels **(A, B)** are originally from Kendall, G.C. et al., 2018 (157). Panels **(C-E)** are originally from Watson, S., LaVigne, CA et al., 2023 (38).

Zebrafish are vertebrates, have high genetic conservation to humans, and are a rapid model to perform functional validation and study of novel RMS fusion oncogenes (154, 155). Previous work has utilized transgenic zebrafish models for PAX3::FOXO1 RMS and infantile/pediatric VGLL2::NCOA2 RMS. Generated tumors recapitulate the human RMS disease transcriptionally and histologically. In the zebrafish PAX3::FOXO1 tumor model, the effect of many promoters/restricted cell lineages was investigated to understand transformation capacity on a broader scale. The *PAX3::FOXO1* sequence and a viral 2A fluor of GFP was integrated with the promoter sequences using the Gateway cloning approach to the Tol2 transposon backbone (156) (**Figure 3A**). This allows for expression on the same mRNA but translation of GFP and PAX3::FOXO1 as independent proteins. These constructs were injected at the single-cell stage of zebrafish embryos and are mosaically but stably integrated into the zebrafish genome. Injected zebrafish were then monitored for tumor formation using phenotypic analysis and GFP expression as a proxy for fusion oncogene expression.

Two promoters that supported transformation included the β-actin promoter and CMV promoter. β-actin driven *PAX3::FOXO1* tumors were consistent with primitive neuroectodermal tumors and did not require a *tp53* mutation to form tumors. On the other hand, CMV-driven *PAX3::FOXO1* tumors required a *tp53* missense mutation (158) to generate rhabdomyosarcoma that recapitulate the human disease (**Figure 3B**). Using this PAX3::FOXO1 zebrafish tumor model, this group also identified a novel PAX3::FOXO1 target gene *her3*, the human ortholog *HES3*, that is upregulated in fusion positive RMS patients (157). They investigated the functional role of *her3*/*HES3* in neural development utilizing the zebrafish model and generated a stable *her3* zebrafish knockout. RNA-seq analysis of the transcriptome suggested that *her3* loss resulted in impairment in organ development and in matrix metallopeptidase function, along with regulating genes involved in apoptosis of the nervous system during development (159). Thus, zebrafish as a model system offers complementary advantages to study transformation capacity and genetic cooperation of PAX3::FOXO1 in RMS.

#### 4.2.5 VGLL2::NCOA2 zebrafish model

VGLL2::NCOA2 is also transforming in zebrafish (38). In this transgenic zebrafish system, the CMV promoter drives expression of the *VGLL2::NCOA2* human coding sequence. This fusion oncogene is linked to a GFP viral 2A sequence, allowing translation as independent proteins. The entire construct is then integrated into the zebrafish genome with the Tol2 mRNA and injection at the single-cell stage (156), and GFP expression (a proxy for fusion oncogene expression) is detected by 24 hours post fertilization. In a wildtype genetic background, 20% of the injected zebrafish developed tumors after 50 days and this percentage increased to 30% after 6 months. This highlights that the VGLL2::NCOA2 fusion oncogene does not require secondary cooperating genes and is sufficient for transformation *in vivo* (**Figures 3C-E**). Generated tumors histologically and transcriptionally recapitulate the human disease. VGLL2::NCOA2 expression led to repression of skeletal muscle differentiation, reactivation or inappropriate persistence of developmental genes, and the model identified potential druggable targets, such as the small GTPase ARF6. Further, ARF6 is overexpressed in VGLL2::NCOA2 driven zebrafish, mouse allograft, and patient tumors compared to normal mature skeletal muscle. Mechanistically, *Arf6* knockout in C2C12 mouse myoblast cells alleviated the VGLL2::NCOA2 differentiation block and suppressed VGLL2::NCOA2 driven colony formation. This supports the rationale to further explore ARF6 as a therapeutic target for VGLL2::NCOA2-driven rhabdomyosarcoma and highlights the zebrafish system as a model to understand new biology.

## 5 Concluding remarks

**Figure 4:**
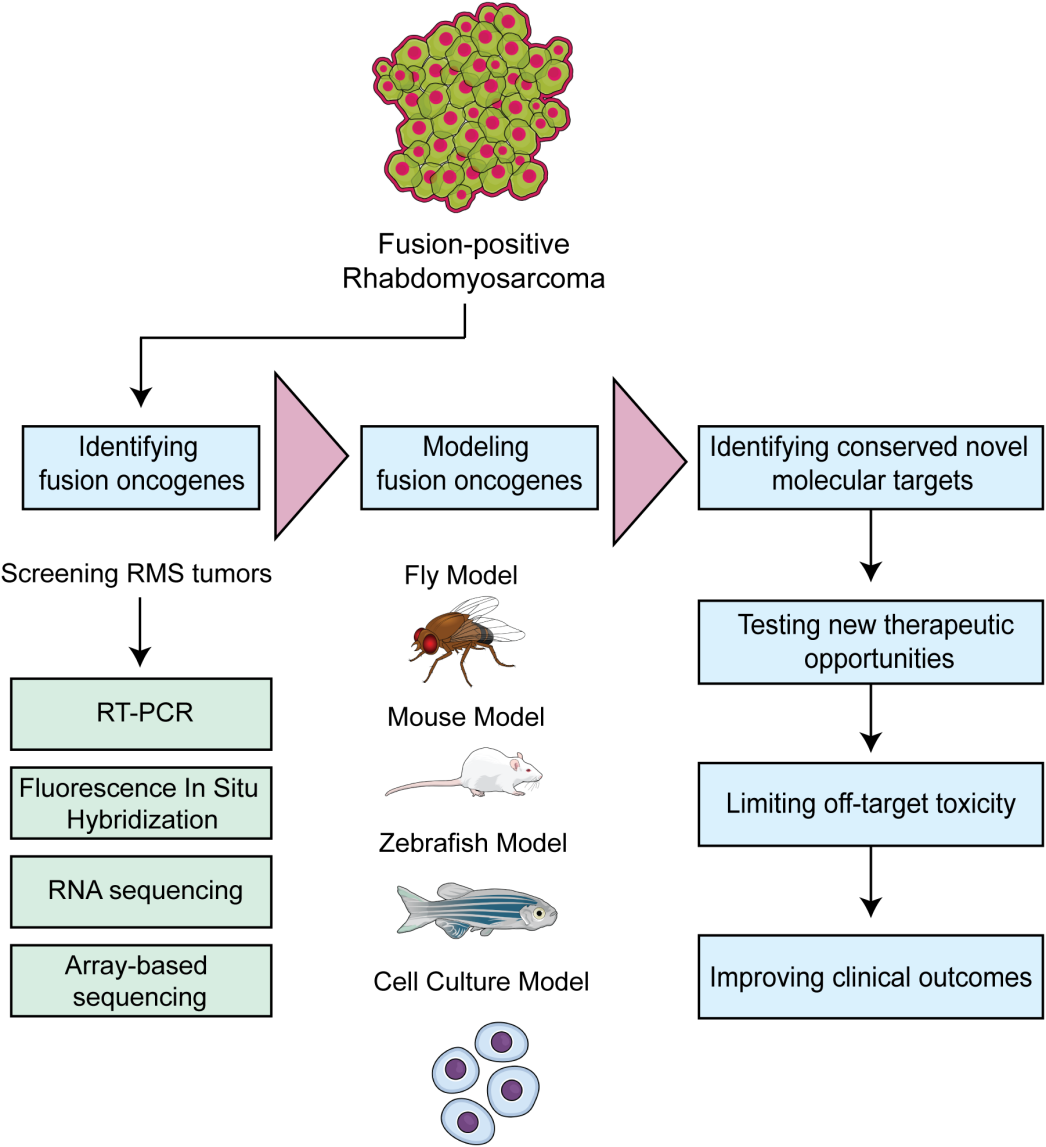
Workflow depicting strategies to model fusion oncogene driven RMS. The approach is to rapidly translate identified fusion oncogenes into tractable model systems and leverage the underlying biology to identify new therapeutic opportunities.

In this review, we highlighted fusion oncogene-driven RMS as a major clinical and scientific challenge. Since they are often the most aggressive RMS subtype, it is critical to understand fusion oncogene biology to identify new molecular targets. We outlined different fusion oncogenes identified in RMS tumors and summarized the research studies that have been performed. The PAX3::FOXO1 fusion is the most common fusion found in alveolar RMS patients, and there have been more studies than in other RMS fusions. In comparison, NCOA2-based fusion oncogenes are newly identified and more effort is needed to understand their biological basis for tumorigenesis. We highlight with the increase in genomic characterization of tumors, there will be an increase in newly identified fusion oncogenes. Therefore, there is a need to functionally validate novel fusion oncogenes using animal and cell culture models, with the anticipation that these models will highlight key biology and therapeutic opportunities. Fusion oncogene expression can also be induced in cells, and these genetically modified cells can then be used in conjunction with animal models to generate tumors that recapitulate the human disease. Tumors can be analyzed for skeletal muscle and RMS protein markers and sequenced using approaches such as RNA sequencing, ChIP sequencing and assay for transposase-accessible chromatin (ATAC) sequencing. Such studies allow for more in-depth understanding of the tumor’s transcriptomic and epigenomic landscape. A challenge is understanding the correct cell type(s) to express the fusion in, or if there is convergent biology across multiple cell types that highlights core mechanisms of fusion oncogene activity. The experimental studies described in this review have increased our understanding of RMS biology along with establishing a platform for future studies. Our proposed workflow for studying fusion oncogene RMS, outlined in **Figure 4**, will determine key molecular targets that can be explored to inhibit tumorigenesis. The overall goal of these systems is to improve therapeutic approaches and clinical outcomes for children with RMS.
